# Language directs spatial attention differently in explicit and implicit tasks

**DOI:** 10.1371/journal.pone.0291518

**Published:** 2023-11-02

**Authors:** Samuel Shaki, Martin H. Fischer

**Affiliations:** 1 Department of Psychology, Ariel University, Ariel, Israel; 2 Department of Psychology, University of Potsdam, Potsdam, Germany; Universiteit Gent, BELGIUM

## Abstract

How do words with either explicit or implicit spatial meanings (e.g., DOWN, BOOT) shift our attention? Recent studies, presenting prime words followed by probe targets, suggested that, for implicit spatial words, both the spatial meaning of prime words and the target locations must be processed to induce congruency benefits. Here we examined the functional necessity of the latter location component. 91 healthy adults discriminated target letters that followed explicit or implicit spatial words. Words either did or did not have to be semantically processed. Target discrimination speed was used to compute congruency benefits. With explicit prime words, spatial congruency effects emerged without semantic processing instructions. In contrast, with implicit prime words, only instructing their semantic processing ensured a congruency benefit. This shows that, for implicit spatial words, spatial processing of target locations is not necessary; instead, processing the spatial connotation of the prime, together with the identity of the target, can induce congruency benefits. Our results help to understand previous conflicting findings.

## Introduction

At the heart of evolutionary success lies the ability to rapidly and accurately perceive, recognize, and respond to objects. Several basic attentional mechanisms support such efficient perception and action, including the orienting response and the rapid fight-flight evaluation mechanism [1]. Interestingly, the same attentional mechanisms also operate in higher cognition, such as the interpretation of symbols, such as directional arrows which explicitly shift attention towards objects or locations.

Attending to objects and their locations improves our perception and recognition of these target objects, even if no overt movements of the eyes or head are involved. Measuring such covert attention allocation typically relies on the successive presentation of an arrow cue and a target stimulus [2, 3]. The arrow cue is meant to prime the observer’s attention by directing it spatially (e.g., to the left or right side); and the target is then shown to measure the observer’s resulting state of attention.

The attention measurement consists of comparing the speed and/or accuracy of either detection or discrimination or other behaviour in response to the target in two experimental conditions: One in which the cue (or prime) directs the observer’s attention to the correct target location (so-called valid or congruent condition); and another where attention is directed elsewhere and needs to be re-directed to apprehend the target at a different location (so-called invalid or incongruent condition). This latter condition typically leads to poorer detection or discrimination performance when compared to the former, indicating a spatial congruency benefit. However, the time between cue onset and target onset (the stimulus onset asynchrony or SOA) can modulate the congruency benefit [4–6].

Not surprisingly, similar results obtain when explicit spatial words like LEFT or RIGHT instead of directional arrows are used as attention cues. In both cases, symbols must be interpreted to understand their meaning in order to affect attentional processing of the targets [7–9]. However, when prime words are only implicitly associated with space, the evidence for similar spatial cueing benefits is mixed. For example, in some studies prime words denoting concrete objects, such as EAGLE and SNAKE, induced vertical congruency effects [10], as did abstract concepts associated with space, such as GOD or SATAN [11]. Other studies found conflicting evidence, showing either facilitation or no effect or interference in congruent conditions [12–14].

In a recent study, Shaki and Fischer [15] addressed this conflict by systematically comparing the effect of processing depth for explicit and implicit spatial words on the spatial congruency effect. The method involved central presentation of stimuli as well as central collection of responses with a single button, as opposed to spatially distributed stimuli or responses in previous work. The rationale for this go-/no-go method was to eliminate explicit spatial coding of stimulus and response locations, which would contaminate the cognitive process of interest with extraneous spatial features [16–18]. For the sake of efficient data collection, the rate of go-trials was between 64% and 69%.

In each experiment, participants performed three cognitive tasks according to rules that required different degrees of prime word and target stimulus processing depth. In the shallow processing condition (detection task), participants merely had to detect all targets, regardless of the meaning of the previously presented prime word. In the intermediate processing condition (localization task), participants only responded to targets in one of two vertical locations, thus enforcing spatial processing of targets. Again, this happened regardless of the meaning of the previously presented prime word. In the deep processing condition (semantic task), participants only responded to targets after determining the relationship between prime word meaning and target location, forcing explicit awareness of the spatial congruity relationship between cue and target, regardless of the explicit or implicit spatial nature of the prime word. For example, one of the four blocked response rules was “respond if the word is associated with up and the target letter appears down” (incongruent block). Thus, both semantic processing of the prime word and localization of the target together determined the decision to respond. Furthermore, we also manipulated the stimulus onset asynchrony (SOA) by imposing three delays between primes and targets (400, 600, and 800 ms) to assess the time course of spatial computations in all conditions.

The results were clear-cut: For explicit spatial prime words, equivalent spatial congruency benefits emerged in all tasks, independent of processing depth for either prime word or target. In contrast, for implicit spatial prime words the spatial congruency effect emerged only in the semantic task, which required both deep processing of the prime word and localization of the target. These results held across all SOAs and revealed no evidence for inhibitory processes in semantic cueing of spatial attention. What remained unclear, however, is whether we need both awareness of the spatial association of the prime word and of the target’s spatial location to obtain the spatial cueing benefit. Alternatively, deep processing of the (explicit or implicit) spatial prime together with non-spatial processing of the target might be sufficient for spatial congruency effects to emerge. The present study addressed this key question.

We replaced our requirement to merely localize the targets in both the Localization Task and the Semantic Task of the above study [15] with a new target discrimination requirement: Participants had to discriminate between the letters X and O. While discrimination tasks are generally considered more sensitive to attentional effects [19], the more important aspect of this change is to remove the previous spatial congruity computation from our task instruction. Specifically, an example go-rule for the Semantic Task will no longer be “respond if prime word is up and target is up” but “respond if prime word is up and target is X”. Will we still find a spatial congruency benefit when the target letter appears in the upper compared to the lower location?

We also examined whether the new results depend on the explicit or implicit spatial nature of the prime words and whether they would be modified by the temporal delay between prime and target. Thus, we tested both prime types in separate experiments and also examined three SOAs (400, 600, and 800 ms) to better understand the mechanisms involved in conceptual cueing of spatial attention.

In summary, we compared two new tasks under explicit (Experiment 1) and implicit (Experiment 2) prime word processing (see Table 1): The discrimination task served as a new baseline without spatial processing requirement for the prime words.

**Table 1:** Overview of task instructions.

	Discrimination Task	Semantic Task
Prime Processing	---	Semantic
Target Processing	Discrimination	Discrimination
Example go rule (respond if…)	… target is X	… word means up and target is O

## Experiment 1: Explicit prime words

### Participants

A total of 49 native Hebrew-reading Israeli adults were recruited from the student populations of Ariel, Israel. They were 10 males and 39 females with ages ranging from 19 to 35 years (mean: 23.2 years). Based on the available effect sizes from similar conditions in the meta-analysis [13], and G-Power calculations (Version 3.1.9.2 [20]), the recommended number of participants is approximately 40.

### Stimuli and apparatus

We used four up-associated spatial words (meaning: up, upper, high, and above) and four down-associated spatial words (meaning: down, lower, low, and below). They were presented visually in Hebrew. These words had also been used as directional primes in previous published work (see above). All Hebrew words were 4–5 letters long and shown in black Arial font with 35 point size on white background. Four additional non-spatial words with similar length and frequency in Hebrew (meaning: door, banana, clock, key; [cf. 21]) were used as fillers. Primes were displayed at the centre of the screen, while the targets (letters X or O) appeared centred horizontally and positioned 8º vertically above or below the centre of the display [12]. Responses were made by pressing the space bar of a QWERTY keyboard. All other keyboard keys were covered. The presentation of task instructions, stimuli, event timing and response recording was controlled by Experiment-Builder software [22].

### Design

There were two different tasks, the order of which was counterbalanced across participants. In the Discrimination Task there were 84 trials, divided into two counterbalanced blocks according to the two response rules. Response rules were either “Respond if the letter is X” or “Respond if the letter is O”. The trials of one block reflected the crossing of two cue types (down, up), two target locations (down, up), and three SOAs (400, 600, and 800 ms). In order to achieve about 70% go trials [see 15], these twelve conditions were implemented once as no-go trials (by presenting the discrimination letter that was not mentioned in the instruction) and two and a half times as go trials. We randomly allocated cue words to all conditions, resulting in 42 trials for each target letter.

In the Semantic Task there were 176 trials, divided into four counterbalanced blocks of different response rules, reflecting the crossing of two cue word meanings (up, down) and two target locations (up, down). The choice of target (X or O was randomized. In each block of 44 trials, there were 30 go trials (70%). These go trials consisted of 10 random combinations of cue words and target letters for each of the three SOAs. The 14 no-go trials were also random combinations of these experimental factors.

### Procedure

Each participant was tested individually after providing informed written consent. This research received formal ethics approval from Ariel University ethics board under AU-SOC-SS-20190204-1. The study was performed in accordance with the ethical standards as laid down in the 1964 Declaration of Helsinki and its later amendments. All trials consisted of two successive visual events: a lexical cue at fixation to which participants did not overtly react, and then, following a randomly chosen SOA, a target letter (X or O) to which participants made a decision that depended on the go rule in a go/no-go task (see Table 1).

Each trial was initiated by a central fixation dot presented for 250 ms. Then, the cue word was presented for 250 ms. After the SOA, the target letter (X or O) was presented and remained visible until the participant’s response or 2000 ms had elapsed (also in no-go trials). Reaction time (RT) was defined as the time from target onset until the participant’s response. No feedback was given, regardless of whether the response was correct or a hit, miss, correct rejection or false alarm.

Participants were instructed to quickly and accurately make a decision according to the response rule (see Table 1) by pressing the space bar. All participants worked under all response rules in a counterbalanced order, always beginning with eight practice trials which were not analysed. As mentioned above, a given participant saw randomly chosen exemplars of each cue type in each condition of our design.

### Analysis

A total of 49 participants were tested but four of them had more than 10% errors and were excluded from analyses. Error trials ranged only from 0% to 8.8% (mean: 1.8%) across the remaining 45 participants so that none was excluded and no error analyses were deemed necessary. Finally, RTs for go trials outside of the mean and 2.5 standard deviations from the group mean were also excluded from analyses (245 trials). The RTs of all remaining trials were averaged across Target Identity (X, O) and analysed with a three-way repeated-measures analysis of variance (ANOVA) that evaluated the effects of 2 Task Instructions (discrimination, semantic; see Table 1), 2 Congruency Levels (congruent: prime means down, target location down; prime means up, target location up; incongruent: prime means down, target location up; prime means up, target location down) and 3 SOAs (400, 600, and 800 ms) on RT. Results were computed with SPSS Version 29.

## Results and discussion

There was a significant main effect of Task Instruction, F(1, 44) = 9.635, p < .005, ηp^2^ = .180. Average RT for the discrimination and semantic tasks were 492 ms and 469 ms, respectively. Why was the discrimination task slower than the semantic task? Our previous observation of a similar disadvantage in the “easy” detection task [15] was attributed to the necessary presence of catch trials. Here, we had no need for catch trials in the discrimination task but again wanted to obtain more than 50% go-trials for the sake of efficient data collection. This led to response-relevant prime words being somewhat predictive of the appearance of response-relevant targets in the semantic task. The resulting response preparation probably explains the speed advantage of the seemingly “harder” task.

There was a reliable main effect of Congruency, F(1, 44) = 32.295, p < .001, ηp^2^ = .423. Average RT for congruent and incongruent conditions was 470 ms and 491 ms, respectively. This result extends the congruency benefit observed by Shaki and Fischer [15] from a detection to a discrimination task. There was also a reliable main effect of SOA, F(2, 88) = 44.887, p < .001, ηp^2^ = .505, with mean RTs of 500, 471, and 469 ms, respectively. Simple main effects showed that the longest SOA yielded reliably faster responses (p < .001) than the two shorter SOAs, which did not differ from each other (p = 1). This pattern probably reflects an aging foreperiod effect, with target appearances becoming more likely with longer SOAs [cf. 23].

**Fig 1:**
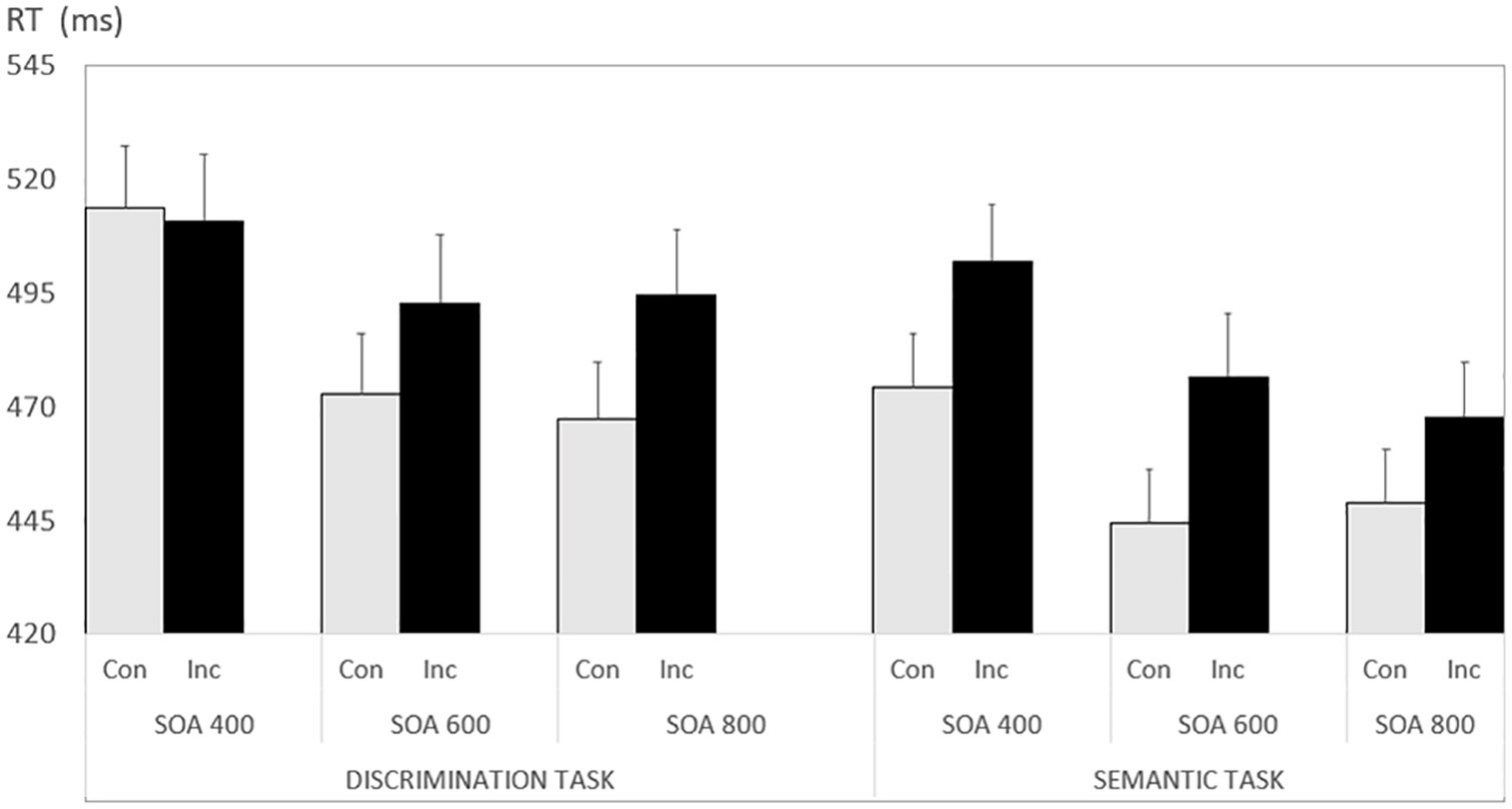
Results of Experiment 1 (explicit prime words). Error bars reflect 1 SEM. For Task descriptions see Table 1.

The only other reliable effect (all other p values > .10) was the triple interaction of all factors, F(2, 88) = 3.237, p < .005, ηp^2^ = .069. As can be seen in Fig 1, this result reflects a lack of congruency effect for the shortest SOA in the discrimination task (paired t-test, p = .384), while all other conditions showed a reliable congruency benefit (all p-values < .008). We interpret this finding as reflecting deeper processing of the spatial meaning of prime words. This interpretation matches our explanation for the overall advantage of the semantic over the discrimination task.

## Experiment 2: Implicit prime words

As expected, Experiment 1 found evidence for the typical spatial facilitation effect through the use of explicit spatial prime words in a discrimination task, thereby replicating and extending the results of Shaki and Fischer [15] that explicit spatial prime words generate spatial attention shifts even without instructed processing of those primes. In slight deviation from that previous work, our SOA manipulation here revealed a slower emergence of the congruency effect with the shallow processing of prime words in the pure target discrimination task. Experiment 2 asked whether this pattern of results would extend to implicit spatial prime words.

### Participants

We recruited 52 new participants from the same population for the second experiment. They were 6 males and 46 females with ages ranging from 20 to 28 years (mean: 23.1 years).

### Stimuli and apparatus

The stimulus set consisted of four Hebrew words associated with upper locations (meaning: hat, roof, cloud, peak) and four Hebrew words associated with lower locations (meaning: carpet, floor, basement, roots). These words were taken from the study by Shaki and Fischer [15]. All other characteristics of stimuli and apparatus were identical to Experiment 1.

### Design and procedure

These were identical to Experiment 1, including the counterbalancing of task and condition blocks.

### Analysis

A total of 52 participants were tested but six had error rates above 10% and were excluded from analyses. These error trials ranged only from 0% to 9.8% across the remaining 46 participants. Finally, RTs for go trials outside of the mean and 2.5 standard deviations were excluded (159 trials). The remaining trials were averaged across Target Identity (X, O) and analysed with the same ANOVA design as before.

## Results and discussion

There was a significant main effect of Task Instruction, F(1, 45) = 6.151, p < .018, ηp^2^ = .120. Average RT for the discrimination and semantic tasks were 457 ms and 474 ms, respectively. This reflects a regular ordering of means with slower performance in the more demanding task.

There was also a reliable main effect of Congruency, F(1, 45) = 19.337, p < .001, ηp^2^ = .301. Average RT for congruent and incongruent conditions was 459 ms and 473 ms, respectively. This result replicates our main finding from Experiment 1 with implicit primes.

Finally, there was a reliable main effect of SOA, F(2, 90) = 31.860, p < .001, ηp^2^ = .415, reflecting again an aging foreperiod effect [cf. 23] with mean RTs of 482, 450, and 465 ms, respectively. Simple main effects showed that the shortest SOA yielded reliably slower responses (p < .001) than the two longer SOAs, which did not differ from each other (p = .168).

**Fig 2:**
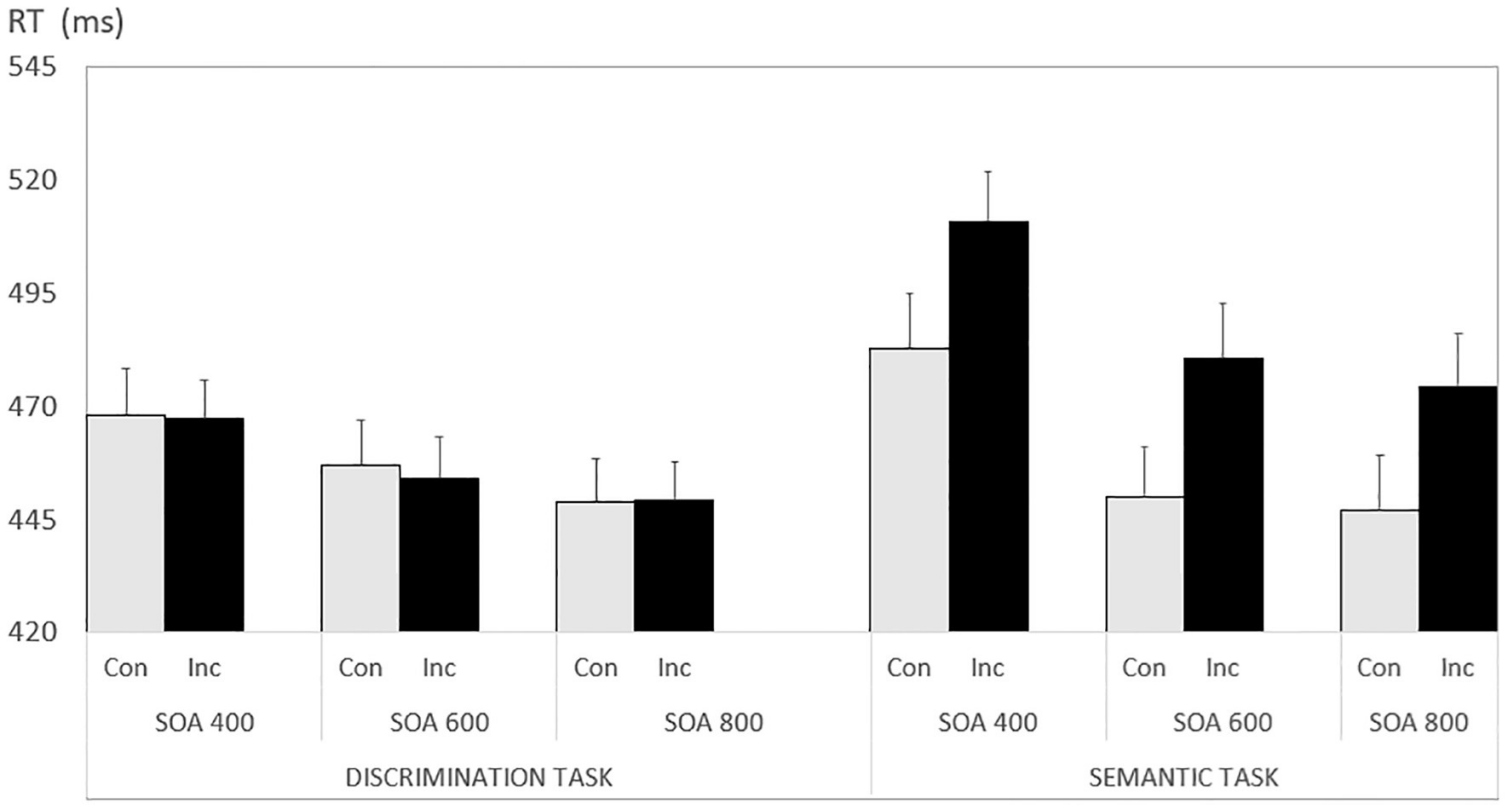
Results of Experiment 2 (implicit prime words). Error bars reflect 1 SEM. For Task descriptions see Table 1.

There were two reliable two-way interactions. First, there was an interaction between Task and Congruency, F(1, 45) = 23.691, p < .001, ηp^2^ = .345. And secondly, there was an interaction between Task and SOA, F(2, 88) = 4.422, p < .015, ηp^2^ = .089. The remaining effects were not reliable (F<1). Both significant interactions can be understood when referring to Fig 2. Specifically, there was no congruency effect across the three SOAs in the discrimination task (all paired t-tests with p > .320), while in the semantic task all SOA conditions showed a reliable congruency benefit (all p-values < .001). This important finding clarifies that, with implicit spatial prime words, congruency benefits only obtain when these primes are semantically processed.

In order to assess possible differences across materials, we also conducted a between-experiment comparison, examining the effects of word type (Experiment 1: explicit spatial words; Experiment 2: implicit spatial words), Task (discrimination, semantic), Congruency (congruent, incongruent) and SOA (400, 600, 800 ms) on RT.

Average RT was 473 ms. There was a reliable main effect of congruency, F(1, 89) = 51.60, p < .001, np^2^ = .367. There was also a significant main effect of SOA, F(2, 178) = 75.81, p <, 001, np^2^ = .46. There was a significant interaction between task and congruency, F(1, 89) = 20.08, p < .001, np^2^ = .184, indicating that the congruency effect depends on processing depth. Importantly, these two factors interacted with word type, F(1, 89) = 3.88, p = .052, np^2^ = .042, signalling that the processing-dependent congruency effect differs for explicit compared to implicit spatial words. Specifically, only when discriminating probes following implicit spatial words there is no congruency effect (see Fig 2). In that same condition, the SOA effect is reduced compared to all other conditions, as indicated by a significant triple interaction of task, SOA and word type, F(2, 178) = 3.09, p = .048, np^2^ = .034. All other effects failed to reach significance, with p-values > .13.

## General discussion

The present study set out to clarify how spatially associated words direct our attention in space. Reading explicit spatial words was known to reliably induced attention shifts in the direction consistent with the word’s meaning, leading to processing benefits for subsequently presented targets in spatially congruent locations. In contrast, implicit spatial words previously produced inconsistent findings. Shaki and Fischer [15] suggested that a necessary requirement for congruency benefits with implicit spatial words is to make the spatial congruency between prime and target explicit by spatially processing both components: The spatial connotation of the prime and the location of the target.

The current work goes beyond this insight by documenting that spatial processing of the target location is not necessary but processing the spatial connotation of the prime, together with the identity of the target is sufficient to induce the congruency benefit. Although the critical interaction for testing the effect of explicit vs. implicit word type on the spatial congruency effect was only marginally significant in a direct statistical comparison, future work should resolve this limitation in statistically stronger designs.

Moreover, we found under explicit processing conditions (Experiment 1) that the time course for the unfolding of the congruency effect differs between tasks. Specifically, there was no congruency effect for the shortest SOA in the discrimination task while all other conditions (including the semantic task) showed a reliable congruency benefit. This observations is in line with work by Davis and Gibson [12] who showed that the available time contributes to the magnitude of the congruency effect in a similar explicit task. Our result clarifies that this time dependence only occurs when participants are not forced to process the spatial meaning of the word cue.

Our present result contributes to the recent debate between Estes and Barsalou [13] vs. Petrova et al. [14] about the presence of interference or facilitation in semantic cueing paradigms. While Estes and Barsalou [13] postulated the presence of interference effects under specific conditions, Petrova concluded from a survey of several experiments (mostly from her own lab) that there is no cueing effect of lexical cues. Our observation of reliable facilitation clarifies that the processing demands of both the cue and the target must be taken into consideration when predicting lexical cueing effects. Specifically, we document that, without semantic processing of the cue, there is no facilitatory effect in congruent conditions [see also 15].

Given that there was only a facilitatory effect and no interference in our paradigm, the interesting question arises whether this facilitatory effect would survive when the task requires only cue discrimination but no deep target processing, e.g. a mere detection task. A previous study [18] had asked participants to discriminate a centrally presented cue (bar vs. arrow) and to respond to a subsequently presented target letter display only following the arrow cues. They found a strong cue validity effect regardless of the instruction to ignore the arrow’s direction. However, this task actually required two successive discrimination processes (first on the cue and then on the letter: H vs U) and therefore does not provide a clear answer to our question. We suggest that further research should address this gap in the literature on semantic cueing benefits by combining a cue discrimination with a target detection task. We should note that our earlier study had also used both X and O as targets but did not require participants to distinguish between them as part of their tasks. Now this methodological similarity between studies [see also 12, 24] facilitates comparisons between results. We again found no evidence for inhibitory effects in the congruent condition, contrary to Estes and colleagues [12, 13, 24]. These diverging outcomes are therefore not due to the attentional demands of target processing but likely reflect the absence of context words in our approach [for details, see 15].

In conclusion, the present study shows that the critical ingredient for conceptual cueing of spatial attention is the semantic activation of spatial meanings in the primes. This happens with explicit spatial primes but should be instructed for implicit spatial primes because otherwise their spatial connotations may or may not become effective, leading to conflicting results. More generally, our results contribute to a more refined understanding of the mechanisms involved in conceptual cueing of spatial attention.
